# Risk Factors of Residual Obstructive Sleep Apnea After Adenotonsillectomy in Children: Systematic Review

**DOI:** 10.3390/medicina62030436

**Published:** 2026-02-26

**Authors:** Paulina Stockunaite, Gintare Oboleviciene, Valdone Miseviciene, Vaidotas Gurskis

**Affiliations:** Pediatric Department, Medical Academy, Lithuanian University of Health Sciences, LT-50103 Kaunas, Lithuaniavaldone.miseviciene@kaunoklinikos.lt (V.M.); vaidotas.gurskis@lsmu.lt (V.G.)

**Keywords:** residual obstructive sleep apnea, children, adenotonsillectomy, risk factors, obesity, Down syndrome

## Abstract

*Background and objective:* Obstructive sleep apnea (OSA) is a common pediatric sleep disorder, most often caused by adenotonsillar hypertrophy. Although adenotonsillectomy (AT) is the standard first–line treatment, a substantial proportion of children experience residual OSA (rOSA). This systematic review aimed to synthesize current evidence on risk factors associated with rOSA in children following AT. *Materials and Methods:* A systematic review was conducted in accordance with PRISMA guidelines. PubMed and the Cochrane Library were searched without date restrictions using English–language terms related to rOSA, children, and adenotonsillectomy. Studies assessing postoperative persistence of OSA and associated risk factors were included. *Results:* Thirteen studies published between 2010 and 2024 met the inclusion criteria. The reported prevalence of rOSA varied widely (18.6–85.0%), reflecting heterogeneity in study design, patient populations, baseline disease severity, and follow–up methods. Obesity emerged as the most consistently identified risk factor, with significantly higher rOSA rates reported among children with elevated body mass index. Age also influenced outcomes, with both very young (<3 years) and older (>7 years) children demonstrating an increased likelihood of persistent disease. Comorbid conditions, particularly asthma and Down syndrome, were associated with poorer postoperative improvement. Additional contributors included craniofacial or developmental abnormalities and higher preoperative apnea–hypopnea index. Limited evidence also suggested that socioeconomic and environmental factors may affect postoperative outcomes. *Conclusions:* Residual OSA is common following adenotonsillectomy in children. Obesity, age, and comorbidities are key predictors, underscoring the need for comprehensive preoperative risk stratification and structured postoperative follow–up.

## 1. Introduction

Obstructive sleep apnea (OSA), the most common form within the spectrum of sleep–disordered breathing, affects 2–10% of children [1,2]. It is characterized by recurrent episodes of upper airway obstruction during sleep, causing intermittent hypoxia, hypercapnia, and sleep fragmentation [3,4]. Persistent sleep–disordered breathing in children can result in multiple negative consequences [3,5,6,7]. These may include behavioral and attention problems, increased hyperactivity, learning challenges, slowed growth, and the possibility of long–term cardiovascular complications [3,6,8].

The most frequent etiology of OSA is adenotonsillar hypertrophy, making adenotonsillectomy (AT) the first–line treatment [3]. Although AT usually improves airway patency and sleep quality, up to 40% of patients show residual OSA (rOSA) postoperatively [1,3,8,9]. The persistence of OSA varies across studies due to differences in patient selection, diagnostic thresholds, and follow–up duration [10].

The clinical significance of rOSA is considerable. Children with persistent sleep–disordered breathing may continue to experience neurocognitive impairment, cardiovascular strain, metabolic dysregulation, and reduced quality of life despite surgical intervention [3,8,11]. Moreover, untreated rOSA often necessitates additional therapeutic strategies, such as continuous positive airway pressure (CPAP), anti-inflammatory therapy, orthodontic or craniofacial interventions, or structured weight management programs [8,12]. Early identification of children at increased risk of postoperative persistence is therefore essential to enable appropriate perioperative counseling, targeted postoperative monitoring, and timely implementation of adjunctive treatments.

Several factors have been implicated, including obesity, age, preoperative OSA severity, comorbidities such as asthma or craniofacial abnormalities, and genetic syndromes like Down syndrome (DS) [6,13,14,15,16]. In addition, social and environmental factors, including passive smoke exposure and socioeconomic status, have been linked to both OSA development and poorer postoperative outcomes [1]. Considering the substantial proportion of children who continue to exhibit OSA following surgical intervention, identifying the factors that contribute to rOSA is essential.

Previous reviews have largely focused on overall treatment outcomes of adenotonsillectomy or on management strategies for rOSA rather than specifically synthesizing predictors of residual disease [12,15]. Furthermore, emerging evidence published in recent years has expanded understanding of metabolic, inflammatory, craniofacial, and social determinants that may influence postoperative outcomes [17,18,19]. Given the heterogeneity in reported rOSA rates and the variability in definitions and follow–up practices, a structured and updated synthesis of risk factors is warranted.

Therefore, this systematic review aims to identify and synthesize current evidence on risk factors associated with rOSA in children aged 1–17 years following adenotonsillectomy, with the objective of supporting improved risk stratification and postoperative management in clinical practice.

## 2. Materials and Methods

This review was performed based on the PRISMA guidelines (Preferred Reporting Items for Systematic Reviews and Meta–Analysis) and was registered in the PROSPERO international prospective register of systematic reviews (Registration No.: CRD420261297160).

### 2.1. Sources of Information and Data Search Strategy

A systematic literature search was conducted from 21 October 2024 to 16 December to identify relevant articles in the PubMed and Cochrane Library databases. These dates refer to the period during which the search was performed. No publication date restrictions were applied. The search of the PubMed and Cochrane Library databases was conducted in English using the following Medical Subject Headings (MeSH) terms and free-text keywords to refine the search strategy: (“residual” [All Fields] OR “residuals” [All Fields]) AND (“obstructive sleep apnea” [All Fields] OR “sleep apnea, obstructive” [MeSH Terms] OR (“sleep” [All Fields] AND “apnea” [All Fields] AND “obstructive” [All Fields])) AND (“child” [MeSH Terms] OR “child” [All Fields] OR “children” [All Fields]) AND (“adenotonsillectomy” [All Fields] OR “adenotonsillectomies” [All Fields]). Further details are available in Supplementary Material Table S1.

### 2.2. Eligibility Criteria

The systematic review included studies that assessed children aged 1 to 17 years who were diagnosed with OSA and underwent AT. Only studies that conducted a follow–up evaluation after surgery using polysomnography (PSG) were included to ensure objective and comparable assessment of rOSA across studies. The exclusion criteria included all studies that evaluated children who did not undergo both AT and post–operative PSG, as well as studies in which rOSA was assessed following treatments or procedures other than AT.

### 2.3. Data Handling

The article selection process followed a four–stage approach to ensure a thorough and systematic review. During the identification stage, duplicate searches were performed to eliminate redundant records. In the screening stage, articles were assessed based on their titles and abstracts, with those not meeting the inclusion criteria or meeting exclusion criteria being removed. The eligibility assessment involved a full–text review, during which studies evaluating other surgical treatment methods or not assessing rOSA after AT were excluded. Only studies that utilized PSG to evaluate rOSA following AT were included in the systematic review.

**Figure 1:**
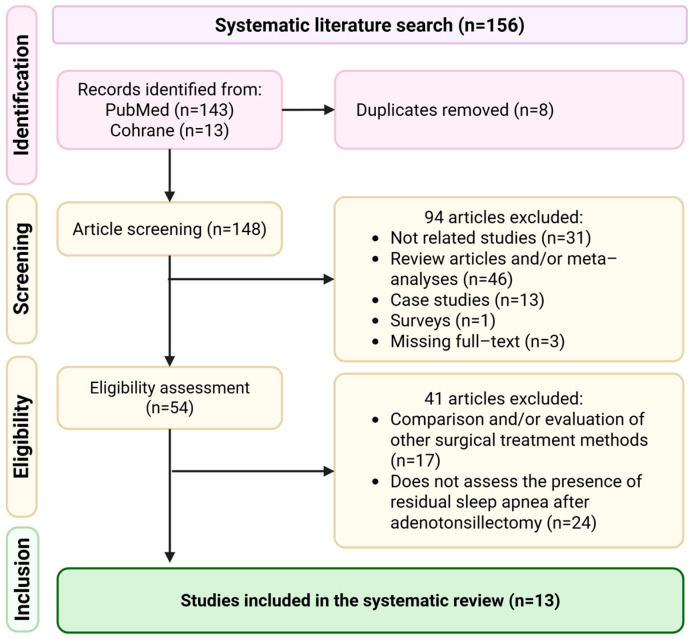
Study selection process from initial search to the final number of included studies.

The systematic review selection process is summarized in the PRISMA flow diagram guidelines (Figure 1). The title and abstract screening resulted in the exclusion of 94 articles based on the inclusion and exclusion criteria. The eligibility assessment was performed on 54 full–text articles, leading to the exclusion of 41 studies. In total, 13 studies were included in the systematic review.

### 2.4. Study Selection and Data Extraction

Two reviewers independently screened titles and abstracts of identified records. Full-text articles of potentially eligible studies were subsequently assessed independently by both reviewers according to predefined inclusion and exclusion criteria. Disagreements were resolved through discussion and consensus.

Data were extracted using a standardized data extraction form developed for this review. The following information was collected from each study: author, year, country, study design, sample size, participants’ age, timing of postoperative PSG, definition of residual OSA (including AHI/OAHI thresholds), reported prevalence of rOSA, analyzed risk factors, and key findings.

Residual OSA was defined according to the AHI/OAHI thresholds used in each individual study. Although most studies applied broadly similar diagnostic criteria (typically AHI > 1 event/hour), minor variations were present. One study used an AHI threshold of >1.5 events/hour, two studies defined rOSA using OAHI > 1 event/hour, and one study applied an OAHI > 2 events/hour cut-off. These differences in diagnostic thresholds were noted across studies.

### 2.5. Data Synthesis

Due to heterogeneity in study populations, outcome definitions, and follow-up protocols, a quantitative meta-analysis was not performed. Findings were synthesized narratively, with extraction of reported prevalence rates of rOSA and identified risk factors. Only the most comprehensive dataset was included.

### 2.6. Quality Assessment

The methodological quality and risk of bias of the included cohort studies were assessed using the Newcastle–Ottawa Scale (NOS). Each study was independently evaluated according to predefined criteria. The overall methodological quality was high (Table 1). The majority of studies were rated as good or very good, while only one study demonstrated moderate quality based on NOS scoring. Detailed scoring results are presented in Supplementary Material Table S2.

## 3. Results

This systematic review analyzed 13 studies, all examining the prevalence and risk factors of rOSA in children after AT. The studies were published between 2010 and 2024. The studies varied in design and methodology, including retrospective and prospective analyses, randomized trials, and multicenter collaborations. The main characteristics of the included studies, such as participants’ age, the interval between AT and reassessment for rOSA, the reported prevalence of rOSA, the risk factors analyzed, and the key study findings, are summarized in Table 2.

The prevalence of rOSA after AT varied widely, ranging from 18.6% to 85.0%, depending on assessment criteria, study population and duration, patients’ characteristics, and the timing of postoperative follow–up.

Among the identified predictors, obesity was the most consistently reported risk factor. Nine studies that analyzed body mass index (BMI) found that children with obesity had markedly higher rates of rOSA, with prevalence ranging from 23.0% to 72.8% [1,9,10,14,20,22,23,25]. One study evaluated OSA prevalence on the first postoperative night and reported a rate of 85.0% among obese children, reflecting immediate postoperative findings [13].

Age was also identified as a factor associated with postoperative outcomes. Several studies reported that older children were more likely to experience rOSA. However, reported prevalence estimates varied widely across studies and should not be interpreted as age-specific rates [1,22,23,25].

Four different studies highlighted asthma as a condition linked with poorer post–AT improvement, especially when coexisting with obesity or more severe OSA before surgery [1,10,20,22]. However, statistical adjustment for confounding factors varied across studies.

Children with DS also showed a high prevalence of rOSA—typically between 54.0% and 68.0%—even though most showed a measurable reduction in apnea-hypopnea index (AHI) after AT [24,26,27]. However, effect estimates and multivariable adjustment were inconsistently reported.

Other contributing factors included craniofacial or developmental abnormalities, reported in some studies with rOSA rates between 38.0% and 84.6%, as well as a higher preoperative AHI, which repeatedly appeared as a strong predictor of persistent symptoms [1,9,10,21,22].

In addition to clinical characteristics, one study highlighted the role of social and environmental influences, such as exposure to tobacco smoke, lower maternal education, or racial and socioeconomic disparities, which were associated with less favorable post–AT outcomes [20].

## 4. Discussion

OSA in children remains one of the most frequent and clinically relevant sleep–related breathing disorder. While AT is widely recognized as the first–line treatment for pediatric OSA, up to 40% of children may continue to exhibit rOSA after surgery [1,3,8,9]. The continued presence of OSA following surgical intervention underscores its complex and multifactorial pathophysiology, encompassing anatomical, metabolic, inflammatory, genetic, and social determinants.

Obesity is consistently identified as one of the most frequently reported predictors of residual disease after AT. Several studies have confirmed that obese children are significantly more likely to have persistent OSA compared to their normal–weight peers [6,13,14,15]. De et al. reported that 85% of obese children continued to exhibit OSA on the first postoperative night [13]. Importantly, subsequent studies indicate that this persistence is not limited to the immediate postoperative period. Bhattacharjee et al. demonstrated that obesity, particularly when combined with older age and chronic asthma, was strongly associated with rOSA in the longer term, with a prevalence of 72.8% [1]. Consistent with these findings, Hsu et al. observed that although surgical intervention resulted in symptomatic improvement across all weight categories, obese children remained at a significantly higher risk of persistent OSA over time [14]. Recent studies suggest that obesity contributes not only through mechanical airway narrowing but also via increased systemic inflammation and altered neuromuscular control [2,28]. In addition, untreated OSA has been associated with metabolic and endocrine dysfunction mediated by inflammatory and neuroendocrine mechanisms, supporting a bidirectional relationship between OSA and obesity [11]. Taken together, these findings indicate that obesity represents one of the most important risk factors for the development and persistence of OSA. Accordingly, current evidence suggests that weight reduction and lifestyle modification constitute essential components of postoperative management, as even modest weight loss following AT can significantly reduce apnea severity and decrease the likelihood of persistent or recurrent OSA in obese pediatric patients [6,12,15,16,17].

In our results, age appears to be a significant determinant of rOSA after AT. Older age has been consistently associated with an increased risk of rOSA following AT, suggesting that age itself may represent an independent risk factor [1,22,23,25]. Potential contributing mechanisms include progressive airway remodeling and age–related changes in upper airway function, while obesity may further exacerbate this risk in older children [1]. Consistent with these findings, Alonso–Álvarez et al. identified age, obesity, and higher baseline respiratory disturbance index as independent predictors of poorer treatment response [23]. Notably, younger obese children tend to achieve higher cure rates and greater improvements in obstructive AHI than their older counterparts, suggesting that age modifies the influence of obesity on treatment outcomes [25]. At the younger end of the age spectrum, rOSA has also been reported, particularly among children with severe preoperative disease or relevant comorbidities. Nath et al. observed that very young children (<3 years) with severe preoperative OSA frequently exhibited residual disease following AT [22]. These finding consistent with the European Respiratory Society statement by Kaditis et al., which emphasizes that young children with comorbidities or severe baseline OSA remain at increased risk of postoperative residual disease [29]. Overall, both very young children and older children appear to be at heightened risk of suboptimal response to AT. However, the underlying mechanisms may differ, with developmental airway factors predominating in younger children and structural or metabolic factors playing a more prominent role in older children.

Asthma is another frequently reported risk factor for rOSA. Both conditions share similar inflammatory pathways, including eosinophilic infiltration, airway hyperreactivity, and nocturnal hypoxemia [28,30]. Bhattacharjee et al. identified chronic asthma as an independent predictor of postoperative OSA, particularly when combined with obesity or severe baseline disease [1]. Imanguli et al. also reported that asthmatic children had higher residual AHI and poorer postoperative outcomes [10]. These associations may be explained by chronic upper airway inflammation and mucosal edema, which increase pharyngeal collapsibility during sleep [28,30]. However, poorly controlled asthma remains a significant risk factor for persistent OSA, highlighting the need for integrated management of both conditions [5].

OSA and allergic diseases are commonly co–occurring respiratory conditions in children, and their interrelationship has been widely and frequently discussed in the scientific literature. Given the close interaction between the upper and lower airways, attention should also be paid to the high prevalence of allergic rhinitis (AR) in children with asthma [31,32]. AR and asthma frequently coexist as part of the “united airway” concept, and many asthmatic patients have either undiagnosed or suboptimally controlled rhinitis [31,32]. Chronic nasal obstruction and increased airway resistance associated with AR can further promote adenoid hypertrophy, mouth breathing and destabilize upper airway patency during sleep, thereby contributing to rOSA risk [33]. Consequently, inadequate recognition or treatment of AR may partially underlie the poorer postoperative outcomes observed in children with asthma, underscoring the importance of evaluating and managing AR in this population [34].

Children with DS represent one of the highest–risk populations for residual and recurrent OSA after AT. Multiple studies have documented rOSA rates ranging from 50% to 70% in this group [24,26,27,35]. Senthilvel et al. found that although AHI significantly improved after surgery, 54% of DS patients continued to experience moderate–to–severe OSA [24]. Tanner et al. reported similar findings and noted that these children often require additional airway management strategies beyond surgery [26]. The persistence of OSA in DS is largely attributed to characteristic craniofacial morphology, including midfacial hypoplasia, macroglossia, narrowed nasopharyngeal lumen, and pharyngeal hypotonia [27,35]. Furthermore, the high prevalence of obesity in this population exacerbates upper airway obstruction [36]. These findings highlight the importance of early postoperative monitoring, follow–up sleep evaluation, and timely consideration of adjunctive therapies, including continuous positive airway pressure or other treatment modalities.

In our systematic review, craniofacial features emerged as important determinants of postoperative outcomes following AT. Maeda et al. reported that 84.6% of children with mandibular hypoplasia continued to exhibit OSA after surgery, with smaller mandibular dimensions correlating with higher postoperative AHI values [21]. Similarly, Imanguli et al. identified craniofacial and neurological abnormalities as significant risk factors for rOSA [10]. These clinical observations are supported by recent cephalometric and three–dimensional imaging studies, which have consistently demonstrated that mandibular retrusion, increased lower facial height, and reduced upper airway dimensions are key structural contributors to pediatric OSA [18,19]. Collectively, these findings suggest that preoperative assessment of craniofacial anatomy may contribute to more informed surgical decision–making and potentially improved long–term outcomes.

Beyond clinical and anatomical factors, socioeconomic and environmental determinants also play a meaningful role. Fayson et al. demonstrated that Black children from socioeconomically disadvantaged neighborhoods were more likely to have rOSA after AT, independent of obesity or preoperative AHI [20]. Similar findings were reported by Wang et al., who found that lower income, maternal education level, and exposure to household smoke were associated with higher postoperative AHI [37]. These disparities may reflect differences in healthcare access, environmental exposures, and adherence to postoperative follow–up [37]. In addition, secondhand smoke and indoor air pollutants may promote airway inflammation, further increasing the risk of persistent OSA symptoms [3,38]. Overall, these findings underscore the contribution of social and environmental contexts to residual disease and highlight the importance of addressing modifiable non–clinical factors in the comprehensive management of pediatric OSA.

This review has several limitations that should be acknowledged. The literature search was conducted in PubMed and the Cochrane Library, both of which provide extensive coverage of peer-reviewed biomedical and clinical research, including the majority of high-impact journals in sleep medicine. However, restricting the search to these databases may have introduced a degree of selection bias, as studies indexed exclusively in other databases or available as grey literature might not have been identified. Conference abstracts, trial registries, and direct author contact were not systematically pursued. In addition, only English-language articles were included. Another limitation is that only a small proportion of the children included in the reviewed studies were younger than three years of age. Therefore, this population subgroup may not be fully represented. Nevertheless, AT is rarely performed in this age group. Several of the included studies were of limited sample size and the results should be interpreted cautiously, particularly the one evaluating abnormal maxillofacial morphology as a potential risk factor. Furthermore, substantial variability was observed in the timing of postoperative PSG reassessment. In some studies, PSG was conducted more than one year after surgery, which may have reduced the accuracy of assessing the true postoperative impact of AT. In addition, heterogeneity across included studies, particularly regarding patient populations, intervals between AT and postoperative PSG, and variability in AHI/OAHI thresholds used to define residual OSA, may have contributed to the wide range of reported prevalence rates and precluded quantitative meta-analysis. Therefore, the findings were synthesized using a structured narrative approach.

A major strength of this review is its comprehensive and systematic approach, encompassing a broad spectrum of risk factors associated with rOSA. The findings clearly delineate subgroups of children who should undergo postoperative PSG assessment following AT and may meaningfully contribute to the development of future clinical guidelines.

## 5. Conclusions

Residual OSA remains a clinically relevant concern in children after AT, although reported prevalence varies widely across studies due to heterogeneity in study design, definitions, and timing of postoperative assessment. The most consistent evidence supports an association between obesity, major comorbidities such as Down syndrome, and higher baseline OSA severity with increased rates of residual disease. Other factors, including craniofacial characteristics and socioeconomic or environmental determinants, have been reported in some studies but appear less consistently supported and should be interpreted cautiously. Given the heterogeneity across studies, careful postoperative assessment may be particularly relevant in higher-risk populations.

## Figures and Tables

**Table 1:** Summary of Methodological Quality Based on Newcastle–Ottawa Scale Scoring.

Quality Category	NOS Score Range	Number of Studies (%)
Very good	7–8	11 (84.6%)
Good	6	1 (7.7%)
Moderate	5	1 (7.7%)

**Table 2:** Description of studies included in the systematic review.

No	Author, Year, Location, Source No.	Study Method	Sample Size	Children’s Age	Time Interval Between the AT and the Reassessment for rOSA	AHI Threshold	Percentage of rOSA Identified	Analyzed Risk Factors	Conclusions
1	Bhattacharjee R., 2010, Europe and USA [1]	Multicenter retrospective study	578	6–12	1.5–24 months	AHI > 1	72.8	Obesity, age (>7 years), chronic asthma	Residual disease is present in a large proportion of children after AT, particularly among older (>7 year) or obese children.
2	Huang Y.S., 2014, Taiwan, Taipei [9]	Prospective longitudinal study	88	6–12	6, 12, 24 and 36 months	AHI > 1	68.0	BMI, body weight, AHI, the presence of enuresis, allergic rhinitis before surgery, age	This study outlines some risk factors, such as severe pediatric OSA, obesity, and a large increase in BMI after AT, rhinitis, enuresis, and older age for recurrence of OSA.
3	Imanguli M., 2016, USA [10]	Retrospective study	169	1–16	118 days	AHI > 1	38.0	Children with obesity, comorbidities including neurological/developmental/craniofacial abnormalities alone or in combination with asthma, or severe OSA.	Teenagers and children with obesity, comorbidities including neurological/developmental/craniofacial abnormalities alone or in combination with asthma, or severe OSA have a high risk of rOSA.
4	De A., 2017, USA [13]	Prospective study	20	8–17	Same night after surgery	AHI > 1	85.0	Obese children	Obese children undergoing AT for OSA are at increased risk for rOSA on the night of surgery.
5	Hsu W., 2012, The Netherlands, Amsterdam [14]	Prospective study	161	2–18	–	AHI > 1	49.1	Obese children	Although sleep parameters improved in all weight statuses, obese children had a higher incidence of rOSA postoperatively. About half of the underweight children shifted to normal weight status after AT.
6	Fayson S.D., 2023, USA [20]	Secondary analysis of a randomized controlled trial	224	5–9	6 months	OAHI > 1	18.6	Obesity, asthma, smoke exposure, sleep duration, maternal education, maternal health, neighborhood disadvantage, black race	Black race was associated with poorer outcome among nonobese children. Obesity, single–female–headed household, maternal major medical diagnosis (cancer, diabetes, heart diseases) was significantly more common among children in the rOSA group.
7	Maeda K., 2014, Japan, Tokyo [21]	Retrospective study	13	4–6	6 months	AHI > 1	84.6	Abnormal maxillofacial morphology	Persistence of OSA after AT may be partly due to the smaller sizes of the mandible in pediatric patients.
8	Nath A., 2013, USA, Chicago [22]	Retrospective study	70	<3 years	<4 months	AHI > 1	21.0	Age, height, weight, BMI, prevalence of asthma, preoperative AHI	The severity of OSA before AT was a predictor of persisting OSA afterward.Data supports the finding that, although AT leads to a significant improvement in children younger than 3 years, a high proportion of this population will have rOSA.
9	Alonso–Alvarez M., 2015, Spain [23]	Prospective, cross–sectional, multicentre study	23	3–14	–	AHI > 1	43.5	Age, obesity	Age, respiratory disturbance index at diagnosis, and obesity are risk factors for rOSA treatment outcomes at follow–up.
10	Senthilvel E., 2024, Switzerland [24]	Retrospective study	48	<18	83% of the follow–up PSG were performed <12 months	AHI > 1.5	54.0	Down Syndrome	Despite the overall significant reduction of OAHI in children with DS and OSA who underwent AT, there was a residual moderate to severe OSA in about half of the included children.
11	Lee T., 2020, USA [25]	Retrospective study	55	<17	~142 days	OAHI > 2	23.0	Older age, obese children	Despite having the highest rates of obesity and the most severe OSA, younger obese patients performed better following AT, with greater cure rate, overall reduction of OAHI, and decreased need for post–surgical nighttime airway support.
12	Tanner S., 2023, Australia [26]	Retrospective study	100	<18	<10 years	OAHI > 1	68.0	Down Syndrome	This study confirms the high prevalence of residual and recurrent OSA in children with DS and describes a largely non–surgical approach to the management of obstruction in this population after initial upper airway surgery.
13	Thottam P. J., 2015, USA [27]	Retrospective study	230	5–8	3–4 months	AHI > 1	65.0	Down Syndrome	Most of the Trisomy 21 patients with severe OSA had residual symptoms following surgical intervention. There is also an increased risk of post–operative airway intervention and increased length of hospital stay in these patients.

Note: AHI—Apnea–Hypopnea Index, AT—Adenotonsillectomy, BMI—Body Mass Index, DS—Down Syndrome, OAHI—Obstructive Apnea–Hypopnea Index, OSA—Obstructive Sleep Apnea, rOSA—residual Obstructive Sleep Apnea.

## Data Availability

No new data were generated or analyzed in this study. Data sharing is not applicable to this article.

## Supplementary Materials

The following supporting information can be downloaded at: https://www.mdpi.com/article/10.3390/medicina62030436/s1. Table S1. The keywords and search results. Table S2. Newcastle–Ottawa Scale analysis of the cohort studies included.

## Abbreviations

The following abbreviations are used in this manuscript:

AHI	Apnea-hypopnea index
AR	Allergic rhinitis
AT	adenotonsillectomy
BMI	Body mass index
CPAP	Continuous positive airway pressure
DS	Down syndrome
MeSH	Medical Subject Headings
NOS	Newcastle–Ottawa Scale
OSA	Obstructive sleep apnea
PRISMA	Preferred Reporting Items for Systematic Reviews and Meta–Analysis
PSG	Polysomnography
rOSA	Residual obstructive sleep apnea
