# Supplementary material for: Risk Factors of Residual Obstructive Sleep Apnea After Adenotonsillectomy in Children: Systematic Review
Source: Medicina (Kaunas). 2026 Feb 26;62(3):436. doi: 10.3390/medicina62030436 (PMC13027964; doi:10.3390/medicina62030436)
Supplement: Supplementary file 1 [file medicina-62-00436-s001.zip › medicina-4148118-supplementary.pdf]

Table S1. The keywords and search results.

Keywords	Search in database	
	PubMed	Cochrane Library
("residual" [All Fields] OR "residuals" [All Fields]) AND ("obstructive sleep apnoea" [All Fields] OR "sleep apnea, obstructive" [MeSH Terms] OR ("sleep" [All Fields] AND "apnea" [All Fields] AND "obstructive" [All Fields]) OR "obstructive sleep apnea" [All Fields] OR ("obstructive" [All Fields] AND "sleep" [All Fields] AND "apnea" [All Fields])) AND ("child" [MeSH Terms] OR "child" [All Fields] OR "children" [All Fields] OR "child s" [All Fields] OR "children s" [All Fields] OR "childrens" [All Fields] OR "childs" [All Fields]) AND ("adenotonsillectomies" [All Fields] OR "adenotonsillectomy" [All Fields])	A total of 143 records were identified, of which 13 fulfilled the predefined inclusion criteria.	A total of 13 records were identified, of which 3 fulfilled the predefined inclusion criteria. However, all included records were duplicates of articles retrieved from the PubMed database.

Table S2. Newcastle–Ottawa Scale analysis of the cohort studies included.

Author, year, location, source no.	Selection				Comparability	Outcome			Total Score	Quality
	Representativeness of the Exposed Cohort	Selection of the Non-Exposed Cohort	Ascertainment of Exposure	Demonstration That Outcome of Interest Was Not Present at Start of Study	Comparability of Cohorts Based on the Design or Analysis	Assessment of Outcome	Was Follow-Up Long Enough for Outcomes to Occur	Adequacy of Follow-Up of Cohorts		
**Bhattacharjee R., 2010, Europe and USA [1]**	+	-	+	+	++	+	+	-	7	Very good
**Huang Y. S., 2014, Taiwan, Taipei [9]**	+	-	+	+	+	+	+	+	7	Very good
**Imanguli M., 2016, USA [10]**	+	-	+	+	++	+	+	+	8	Very good
**De A., 2017, USA [13]**	+	-	+	+	-	+	+	+	6	Good

**Hsu W., 2012, Netherlands, Amsterdam [14]**	+	-	+	+	++	+	+	+	8	Very good
**Fayson S. D., 2023, USA [20]**	+	-	+	+	++	+	+	+	8	Very good
**Maeda K., 2014, Japan, Tokyo [21]**	-	-	+	+	-	+	+	+	5	Moderate
**Nath A., 2013, USA, Chicago [22]**	+	-	+	+	++	+	+	+	8	Very good
**Alonso – Alvarez M., 2015, Spain [23]**	+	-	+	+	++	+	+	+	8	Very good
**Senthilvel E., 2024, Switzerland [24]**	+	-	+	+	+	+	+	+	7	Very good

**Lee T., 2020, USA [25]**	+	-	+	+	+	+	+	+	7	Very good
**Tanner S., 2023, Australia [26]**	+	-	+	+	+	+	+	+	7	Very good
**Thottam P. J., 2015, USA [27]**	+	+	+	+	+	+	+	+	8	Very good
